# Childhood Exposure to Air Pollution, Body Mass Index Trajectories, and Insulin Resistance Among Young Adults

**DOI:** 10.1001/jamanetworkopen.2025.6431

**Published:** 2025-04-22

**Authors:** Fangqi Guo, Xinci Chen, Steve Howland, Zhongzheng Niu, Lu Zhang, W. James Gauderman, Rob McConnell, Nathan Pavlovic, Fred Lurmann, Theresa M. Bastain, Rima Habre, Carrie V. Breton, Shohreh F. Farzan

**Affiliations:** 1 Department of Population and Public Health Sciences, Keck School of Medicine, University of Southern California, Los Angeles; 2 Sonoma Technologies Inc, Petaluma, California

## Abstract

**Importance:**

Emerging evidence suggests that exposure to air pollution affects children’s glucose metabolism. However, the underlying mechanisms are not fully understood.

**Objective:**

To investigate whether body mass index (BMI; calculated as the weight in kilograms divided by the height in meters squared) growth trajectories mediate the association between traffic-related air pollution (TRAP) and insulin resistance.

**Design, Setting, and Participants:**

As part of the Southern California Children’s Health Study, the ongoing Meta-Air2 cohort substudy followed up participants from pregnancy to 24 years of age and examined the mediation role of BMI. Cardiometabolic follow-up was initiated as participants transitioned into adulthood. Data from the Meta-Air2 substudy were collected from November 27, 2018, to May 31, 2023.

**Exposures:**

The California Line Source Dispersion Model was used to calculate mean childhood exposure to traffic-related total nitrogen oxides (NO_x_) from pregnancy to 13 years of age by calculating mean monthly estimates. Traffic density within a 300-m buffer around participants’ residence was calculated as a secondary outcome.

**Main Outcomes and Measures:**

Insulin resistance markers were assessed during the recent visit in young adulthood, including the homeostatic model assessment of insulin resistance (HOMA-IR; calculated from fasting glucose and insulin levels) and glycated hemoglobin (HbA_1c_) level. Participants’ BMI growth trajectories, including BMI at 13 years of age and accelerated BMI growth, were analyzed as potential mediators. Using fully adjusted PROCESS macro mediation models, their role in mediating the association between traffic-related total NO_x_ exposure and insulin resistance was examined with adjustment for demographic characteristics, smoking status, and parental history of diabetes.

**Results:**

Among the 282 participants (mean [SD] age, 24.0 [1.7] years), each 1-SD increase in childhood exposure to traffic-related total NO_x_ was associated with a BMI increase of 0.71 (95% CI, 0.29-1.13) at 13 years of age and adult HOMA-IR increase of 0.55 (95% CI, 0.23-0.87). An estimated mediation effect identified BMI at 13 years of age combined with accelerated BMI growth as accounting for 41.8% of the estimated total effect (β, 0.23; 95% bootstrap CI, 0.01-0.52) between total NO_x_ and HOMA-IR. Similar patterns were observed when exploring traffic density as an exposure or HbA_1c_ level as an outcome.

**Conclusions and Relevance:**

In this cohort study of young adults, the long-term association between TRAP and insulin resistance may be partially explained by higher BMI and accelerated BMI growth from early adolescence into young adulthood. These findings highlight the importance of weight management in children, particularly those residing in highly polluted areas.

## Key Points

**Question:**

Do body mass index (BMI) growth trajectories mediate the association of air pollution exposure with insulin resistance?

**Findings:**

In this cohort study of 282 participants tracked from pregnancy to 24 years of age, childhood BMI growth trajectories accounted for half of the observed effect estimates of exposure to traffic-related air pollution with insulin resistance among young adults.

**Meaning:**

These findings highlight the importance of weight management in children, particularly those residing in highly polluted areas.

## Teaser

This cohort study assesses whether body mass index growth trajectories mediate the association between traffic-related air pollution and insulin resistance among young adults.

## Introduction

Insulin resistance is a condition in which cells become less responsive to the effects of insulin.^1^ When insulin resistance persists over time, type 2 diabetes (T2D) develops. Although T2D and insulin resistance are more prevalent in older populations, their rising incidence among young adults and children is a growing problem.^2^

Traffic-related air pollutants (TRAP) are a combination of gaseous and particulate matter discharged into the atmosphere from traffic-related emissions.^3^ TRAP exposure has been associated with a higher risk of developing T2D.^4,5^ However, the mechanism underlying this association is not fully understood. Weight status, which is associated with both air pollution exposure^6,7^ and insulin resistance,^8^ may play an important role in this link. In previous studies, weight status has been more frequently examined as an effect modifier rather than a mediator because of the cross-sectional nature of other study designs.^9,10^ However, the onset of overweight can occur in early childhood, far earlier than the typical onset time of insulin resistance. Therefore, childhood exposure to air pollution may directly lead to rapid weight gain, eventually resulting in insulin resistance or glucose dysregulation. Previous studies that examined BMI and glucose regulation simultaneously precluded the possibility of mediation analysis.^9,10^ Testing such a mediation hypothesis requires a longitudinal study with time-lagged measurement of pollution exposure, BMI, and insulin resistance.

The Southern California’s Children’s Health Study (CHS) is one of the largest investigations of the longitudinal impacts of air pollution on children’s health.^11^ Previous CHS studies reported associations between early-life TRAP exposure and accelerated BMI growth in children.^12,13,14^ As investigators, including members of our group, have continued to follow up CHS participants over time, we have extended this investigation of BMI trajectories from early adolescence into young adulthood, and assessed the potential role of BMI growth as a mediator between TRAP and insulin resistance. Specifically, this study aimed to examine (1) the association between childhood exposure to traffic-related nitrogen oxides (NO_x_) and adult insulin resistance, (2) the extent to which BMI growth trajectories mediate the association between total NO_x_ exposure and insulin resistance, and (3) direct association between air pollution exposure, BMI growth trajectories, and insulin resistance.

## Methods

### Data

Our study, Meta-Air2, is an ongoing substudy of the larger CHS Cohort E (eFigure 1 in Supplement 1).^15,16,17^ Participants were enrolled in cohort E in 2003, in kindergarten or first grade and actively followed up until 2014. As these participants transitioned into young adulthood on November 27, 2018, we initiated a follow-up study on a subset of cohort E participants who had undergone cardiometabolic assessments in childhood and were willing to participate in the follow-up assessment. By May 31, 2023, 283 participants had provided blood samples for metabolic biomarker testing. All study protocols and materials were approved by the University of Southern California’s Institutional Review Board. Participants provided written informed consent before their study visit. Our analysis follows the Strengthening the Reporting of Observational Studies in Epidemiology (STROBE) reporting guideline.

### Measures

#### Traffic-Related Total NO_x_

CHS maintains detailed records of each participant’s residential history, geocoded at the monthly level, starting from pregnancy through early adolescence (year 2010, mean [SD] age, 13.0 [0.6] years). Timelines integrated multiple information sources (eg, birth certificates, historical CHS questionnaires). For participants who stayed in more than 1 place within a month, their monthly exposures were time-weighted based on time spent at each location. These timelines formed the basis of all air pollution exposure assessments.

We used the California Line Source Dispersion Model (CALINE4) to estimate residential traffic-related NO_x_ concentrations from nearby on-road vehicles.^3^ Details about CALINE4 are provided in eMethods in Supplement 1. The traffic-related total NO_x_ estimates by road class capture primarily spatial variations in emissions, traffic volume, roadway geometries, and variability in meteorology over time that impact the dispersion of on-road vehicle emissions. Changes in annual emission rate are scaled to the 2002 NO_x_ emissions of the Southern California air basin fleet. The roadway network data were retrieved for all road classes from the Environmental Systems Research Institute Streetmap Premium national database. For our study, we used traffic-related total NO_x_ as a proxy for the overall TRAP mixture because total NO_x_, estimated by CALINE4, is highly correlated with the entire TRAP mixture at the local spatial scale.^18^ Additionally, traffic density was calculated within a 300-m buffer around the residence using annual mean daily traffic volumes as a secondary exposure. Mean childhood air pollutant exposures from pregnancy to early adolescence were determined by calculating mean monthly estimates. Associations with quartiles of exposure to traffic-related total NO_x_ were also examined.

#### BMI

Participants’ weight and height were objectively measured at mean ages of 13, 15, and 24 years. At each time point, weight and height were measured 3 times and the mean was calculated to determine the individual’s BMI, calculated as the weight in kilograms divided by the height in meters squared.

#### Insulin Resistance

The homeostatic model assessment of insulin resistance (HOMA-IR) provides a numeric value to help determine an individual’s level of insulin resistance based on fasting glucose and insulin levels. At the young adult time point (mean [SD] age, 24.0 [1.7] years), trained staff collected blood samples after an overnight fast of 8 to 10 hours. Blood samples were processed into plasma and subsequently delivered to the University of Southern California Metabolic Assay Core for analysis of fasting glucose and insulin levels. HOMA-IR was calculated using the formula HOMA-IR = [insulin (μU/mL)] × [glucose (mg/dL)]/405 to determine participants’ insulin resistance levels. Higher HOMA-IR values indicate greater insulin resistance.

We also investigated participants’ levels of glycated hemoglobin (HbA_1c_) as a secondary outcome. Using a glycemic monitor (DCA Vantage Analyzer; Siemens Medical Solutions USA Inc), whole blood samples were analyzed for HbA_1c_ levels, recorded as percentage.

### Statistical Analysis

By May 31, 2023, 283 participants had provided blood samples for the evaluation of insulin resistance. One participant with an unrealistic fasting insulin value was excluded, resulting in a final analytical sample of 282 participants. For the secondary outcome, HbA_1c_ level, 5 participants lacked sufficient blood for testing, and 1 extreme value was excluded from the analysis, leaving a final HbA_1c_ sample size of 276 participants.

We used a 2-step approach to test the study aims. First, we estimated BMI growth trajectories for each individual from early adolescence to young adulthood using BMI measurements taken at the mean ages of 13, 15, and 24 years (eFigure 2 in Supplement 1). Given that an individual’s BMI growth trajectory from 13 to 24 years of age follows a roughly linear pattern,^19^ we used a mixed-effects linear model to estimate BMI using age while accounting for random age-related variations within each individual. Age was centered at 13 years, with the intercept representing the estimated BMI when participants were 13 years of age and the slope representing linear BMI growth rate. The BMI intercept and growth slope for each individual were extracted for subsequent mediation analyses.

Second, we used Hayes PROCESS macro mediation model 6 to investigate the estimated total effect of air pollution with insulin resistance, as well as the estimated mediation effects of childhood BMI.^20^ This particular model allows multiple mediators operating in serial order. To determine the significance of associations in mediation analysis, the PROCESS macro follows these steps. First, it uses standard linear regression model to test the effect of the independent variable (X) on the mediator (M), adjusting for covariates (path a). Second, it tests the effect of the mediator (M) on the dependent variable (Y), while controlling for the independent variable (X) and covariates (path b). The indirect effect is the product of the path coefficients from X → M (a) and M → Y (b), representing the influence of X on Y through M. The PROCESS macro uses the bootstrap method to generate a CI for the indirect effect. This method involves repeatedly resampling the data and estimating the indirect effect in each resampled dataset. If the entire bootstrap CI lies entirely above zero or entirely below zero, this provides evidence of a statistically significant indirect effect.^20,21^

The exposures in our models were mean childhood exposure to traffic-related total NO_x_ and traffic density, from pregnancy to 13 years of age. Total NO_x_ and traffic density were standardized with a mean of zero and an SD of 1. This approach allowed the analytical coefficients to be interpreted as the impact on an outcome for each 1-SD change in air pollution exposure. The study outcomes were HOMA-IR and HbA_1c_ levels. Mediators included the estimated BMI at 13 years of age (intercept) and accelerated BMI growth (slope). Since 11.7% of measured values for BMI at 13 years of age were missing, we opted to use estimated BMI values at 13 years of age in analyses.

Covariates, including age in young adulthood, race (American Indian or Alaska Native, Asian or Pacific Islander, Black, White, multiracial, other [including Indonesian, Iranian, Latino, Middle Eastern, Persian, Portuguese, Romanian, Slavic, or left the response unspecified], or unknown), ethnicity (Hispanic or non-Hispanic), sex (female or male), participant smoking status in young adulthood (never, former, or current), parents’ educational level, and parental history of diabetes, were adjusted in the mediation modeling (eFigure 3 in Supplement 1). The category of American Indian or Alaska Native was combined with other in the analysis. The parents’ highest attained educational level was used to represent the family socioeconomic status of the participants. Statistical analyses were conducted using Stata SE, version 17.0 (StataCorp LLC), and R, version 4.2.2 (R Program for Statistical Computing).

## Results

Among the 282 study participants, there were comparable numbers of female (144 [51.1%]) and male (138 [48.9%]) as well as Hispanic (149 [52.8%]) and non-Hispanic (133 [47.2%]) individuals. In terms of race, 40 participants (14.2%) self-identified as American Indian, Alaska Native, or other; 15 (5.3%), Asian or Pacific Islander; 4 (1.4%), Black; 172 (61.0%), White; 34 (12.1%), multiracial; and 17 (6.0%), unknown. Most participants reported never smoking (160 [56.7%]). Twenty-three participants (8.2%) reported a parental history of diabetes. Overall, air pollution exposure levels were found to be within expected ranges in Southern California (Table 1).^22^

**Table 1.** Description of Study Sample

Characteristic	No. (%) (N = 282)
Age at adult visit, mean (SD), y	24.0 (1.7)
Sex	
Female	144 (51.1)
Male	138 (48.9)
Race	
Asian or Pacific Islander	15 (5.3)
Black	4 (1.4)
White	172 (61.0)
Multiracial	34 (12.1)
Other^a^	40 (14.2)
Not known	17 (6.0)
Ethnicity	
Hispanic	149 (52.8)
Non-Hispanic	133 (47.2)
Highest level of parental education	
Less than high school	19 (6.8)
High school	38 (13.5)
Some college	93 (33.0)
Undergraduate degree	50 (17.7)
Graduate degree	60 (21.3)
Not reported	22 (7.8)
Smoking status	
Never	160 (56.7)
Former	75 (26.6)
Current	47 (16.7)
Parental history of diabetes	
No	222 (78.7)
Yes	23 (8.2)
Not reported	37 (13.1)
Environmental exposure (aged 0 to 13 y), monthly mean (SD)	
Traffic-related total NO_x_, ppb	17.0 (14.6)
Traffic density (within 300-m buffer)	38.0 (62.7)
BMI change over time, mean (SD)	
Estimated BMI at 13 y of age	20.6 (3.3)
Accelerated BMI growth from 13 to 24 y of age	0.52 (0.42)
Glucose metabolism outcome, mean (SD)	
HOMA-IR (unitless)	1.3 (1.4)
HbA_1c_ level, mean (SD), % (n = 276)	5.3 (0.4)

Abbreviations: BMI, body mass index (calculated as the weight in kilograms divided by the height in meters squared); HbA_1c_, hemoglobin A_1c_; HOMA-IR, Homeostatic Model Assessment for Insulin Resistance; NO_x_, nitrogen oxides; ppb, parts per billion.

SI conversion factor: To convert HbA_1c_ to proportion of hemoglobin, multiply by 0.01.

^a^ Includes American Indian or Alaska Native, Indonesian, Iranian, Latino, Middle Eastern, Persian, Portuguese, Romanian, Slavic, and unspecified.

### Estimated Total, Direct, and Indirect Effects

As displayed in Table 2, a 1-SD increase in mean childhood exposure to traffic-related total NO_x_, which was 18.7 parts per billion (ppb) in our study, was associated with a 0.55 (95% CI, 0.23-0.87) greater adult HOMA-IR. This estimated total effect was composed of 58.2% estimated direct effect from total NO_x_ with HOMA-IR (β, 0.32; 95% CI, 0.05-0.59), as well as 41.8% estimated indirect effects through combined accelerated BMI growth and BMI at 13 years of age (β, 0.23; 95% bootstrap CI, 0.01-0.52). We observed significant indirect mediation through BMI at 13 years of age alone (β, 0.15; 95% bootstrap CI, 0.03-0.29), and the serial mediation from BMI at 13 years of age to accelerated BMI growth (β, 0.05; 95% bootstrap CI, 0.003-0.12). A similar pattern was observed when investigating traffic density as an exposure, as shown in Table 2. In subgroup analyses focusing on female and male participants individually (eTable 1 in Supplement 1), significant estimated mediation effects of BMI growth trajectory were observed among female but not among male participants.

**Table 2.** Estimated Direct, Indirect, and Total Effects of Childhood Exposure to Traffic-Related Air Pollution on Adult HOMA-IR^a^

Exposure	β (95% CI)	*P* value
**Traffic-related total NO_x_**		
NO_x_ to M1 to HOMA-IR^b^	0.15 (0.03 to 0.29)	NA
NO_x_ to M2 to HOMA-IR^c^	0.04 (−0.13 to 0.26)	NA
NO_x_ to M1 to M2 to HOMA-IR	0.05 (0.003 to 0.12)	NA
Total indirect role	0.23 (0.01 to 0.52)	NA
Direct role		
NO_x_ to HOMA-IR	0.32 (0.05 to 0.59)	.02
Total role		
NO_x_ to HOMA-IR	0.55 (0.23 to 0.87)	.001
**Traffic density**		
Density to M1 to HOMA-IR	0.12 (0.02 to 0.24)	NA
Density to M2 to HOMA-IR	0.07 (−0.09 to 0.27)	NA
Density to M1 to M2 to HOMA-IR	0.04 (0.0004 to 0.09)	NA
Total indirect role	0.23 (0.01 to 0.47)	NA
Direct role		
Traffic density to HOMA-IR	0.24 (−0.01 to 0.50)	.06
Total role		
Traffic density to HOMA-IR	0.47 (0.17 to 0.77)	.003

Abbreviations: HOMA-IR, Homeostatic Model Assessment of Insulin Resistance; NA, not applicable; NO_x_, nitrogen oxides.

^a^ Results are from PROCESS macro mediation model 6 (N = 282). Bootstrap CIs were displayed for indirect effects. All analyses adjusted for adulthood age, sex, race, ethnicity, smoking status, parents’ highest degree, and parental history of diabetes.

^b^ M1 represents the estimated individual body mass index (BMI) (intercept) at 13 years of age.

^c^ M2 represents individual accelerated BMI growth (slope) from approximately 13 to 24 years of age.

We also observed a similar pattern when investigating HbA_1c_ level as an outcome (eTable 2 in Supplement 1). We found that a 1-SD increase in mean childhood exposure to traffic-related total NO_x_ was associated with an increase of 0.08 (95% CI, 0.03-0.14) in adult HbA_1c_ levels, and a 1-SD increase in mean childhood exposure to traffic density was associated with an increase of 0.08 (95% CI, 0.03-0.13) in adult HbA_1c_ levels (to convert HbA_1c_ to proportion of hemoglobin, multiply by 0.01). The estimated total mediation effects through BMI were 0.02 (95% bootstrap CI, −0.0004 to 0.06) for traffic-related total NO_x_ and 0.02 (95% bootstrap CI, −0.001 to 0.06) for traffic density.

### Paths Linking Exposure, Mediators, and Outcomes

**Figure 1.**
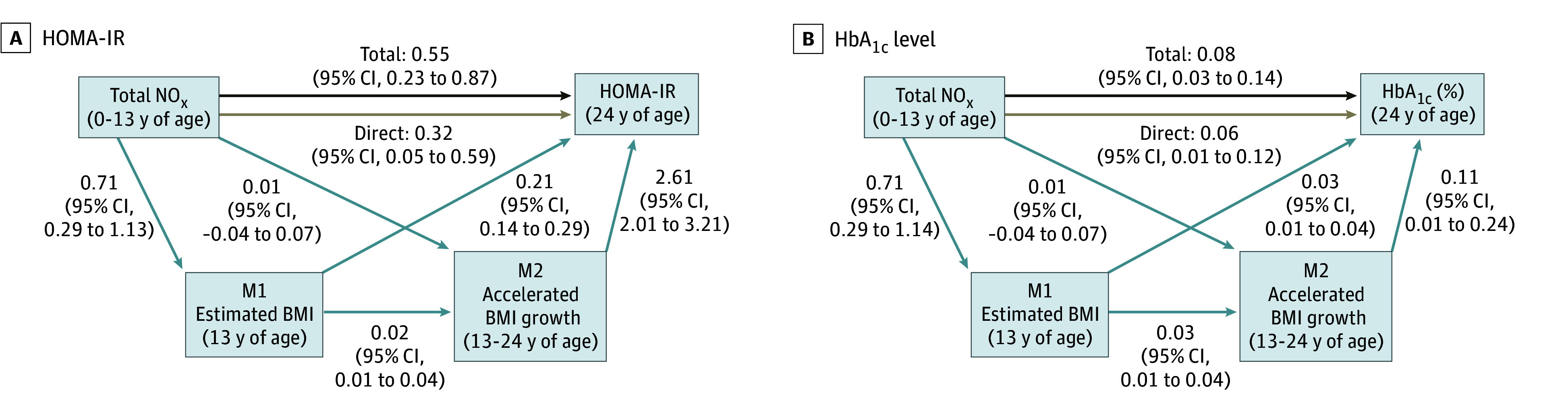
Paths Between Childhood Exposure to Traffic-Related Total Nitrogen Oxides (NO_x_), Body Mass Index (BMI), and Adult Homeostatic Model Assessment of Insulin Resistance (HOMA-IR) and Glycated Hemoglobin (HbA_1c_) All analyses are adjusted for adulthood age, sex, race, ethnicity, smoking status, parents’ highest educational attainment, and parental history of diabetes. β Coefficients and corresponding 95% CIs are included for each tested association.

The direct associations between air pollution exposure and each of the BMI mediators, as well as the direct associations between BMI mediators and each outcome, are displayed in Figure 1 and eTable 3 in Supplement 1. Childhood exposure to traffic-related total NO_x_ (β, 0.71; 95% CI, 0.29-1.13) and traffic density (β, 0.56; 95% CI, 0.16-0.96) were associated with BMI at 13 years of age. Although neither total NO_x_ exposure nor traffic density were associated with accelerated BMI growth, higher BMI at 13 years of age was associated with accelerated BMI growth from 13 to 24 years of age (β, 0.02; 95% CI, 0.01-0.04). Higher BMI at 13 years of age (β, 0.21; 95% CI, 0.14-0.29) and accelerated BMI growth (β, 2.61; 95% CI, 2.01-3.21) were associated with higher levels of HOMA-IR in young adulthood. BMI at 13 years of age was also associated with higher HbA_1c_ levels in young adulthood (β, 0.03; 95% CI, 0.01-0.04).

### Exposure Quartiles and BMI Growth Trajectories

**Figure 2.**
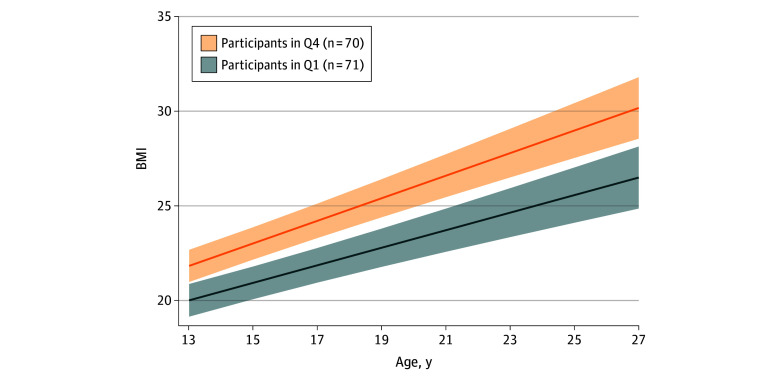
Body Mass Index (BMI) Trajectories Between Children Exposed to Upper and Lower Quartiles of Traffic-Related Total Nitrogen Oxides (NO_x_) BMI is calculated as the weight in kilograms divided by the height in meters squared. BMI growth trajectories are estimated for participants exposed to the fourth (Q4) and first (Q1) quartiles of traffic-related total NO_x_ during childhood (monthly mean exposure from pregnancy to 13 years of age), based on results from a mixed-effects linear model. Shaded areas indicate 95% CIs.

Figure 2 illustrates the BMI growth trajectories between participants who were exposed to the upper quartile (mean [SD], 47.3 [21.3] ppb) and lower quartile (mean [SD], 6.8 [2.9] ppb) of traffic-related total NO_x_ in childhood. Overall, we observed distinct BMI growth trajectories across these 2 groups (Table 3). Children exposed to the highest quartile of total NO_x_ had significantly higher BMI at 13 years of age (mean [SD], 21.9 [4.1] vs 20.0 [2.5]; *P* < .001), higher BMI in young adulthood (mean [SD], 28.4 [8.5] vs 25.1 [5.0]; *P* = .002), higher adult HOMA-IR (mean [SD], 2.8 [3.9] vs 1.4 [1.0]; *P* = .002), and higher HbA_1c_ levels (mean [SD], 5.5% [0.7] vs 5.2% [0.3]; *P* = .004), compared with the lowest quartile of total NO_x_ exposure. The differences remained significant after adjusting for variance within residential communities and covariates (Figure 2). Similar patterns were observed for traffic density.

**Table 3.** Comparison of Key Variables Between Children Exposed to the Upper and Lower Quartiles of Traffic-Related Total NO_x_

Variable of interest	Exposure in childhood, mean (SD)^a^		Differences between groups, *P* value	
	Quartile 4 (n = 70)	Quartile 1 (n = 71)	Independent-samples *t* test	Mixed-effects linear model^b^
Traffic-related total NO_x_	47.3 (21.3)	6.8 (2.9)	<.001	<.001
Traffic density	162.7 (108.8)	20.7 (11.2)	<.001	<.001
Accelerated BMI growth	0.59 (0.5)	0.47 (0.4)	.05	.25
BMI at 13 years of age	21.9 (4.1)	20.0 (2.5)	<.001	.04
BMI at 24 years of age	28.4 (8.5)	25.1 (5.0)	.002	.04
HOMA-IR	2.8 (3.9)	1.4 (1.0)	.002	.003
HbA_1c_ level	5.5 (0.7)	5.2 (0.3)	.004	.009

Abbreviations: BMI, body mass index (calculated as the weight in kilograms divided by the height in meters squared); HbA_1c_., hemoglobin A_1c_; HOMA-IR, Homeostatic Model Assessment for Insulin Resistance; NO_x_, nitrogen oxides.

^a^ Measured as monthly mean from pregnancy to 13 years of age.

^b^ Mixed effects linear model adjusted for random intercept within residential communities and other covariates including age (at time of adult assessment), sex, race, ethnicity, adult smoking status, parents’ highest degree, and parents’ diabetes history.

## Discussion

Our cohort study is among the first, to our knowledge, to use life course data to examine the estimated mediation effect of BMI growth trajectory in the association of air pollution exposure with insulin resistance in young adults. We found that mean childhood exposure to traffic-related total NO_x_ from pregnancy until early adolescence was associated with higher levels of insulin resistance and higher HbA_1c_ levels in young adults around 24 years of age. BMI growth trajectories from early adolescence to young adulthood explained 41.8% of estimated total effect of traffic-related total NO_x_ exposure with HOMA-IR.

Our finding that each 18.7-ppb increase in traffic-related total NO_x_ exposure is associated with a 0.55 increase in HOMA-IR suggested that the impact of air pollution on health is not subtle. Consistent with our findings, Andersen et al^23^ followed up more than 50 000 adults for 10 years and found that long-term exposure to traffic-related NO_x_ was associated with higher risk of diabetes incidence. Additionally, the adverse effects of TRAP on glucose regulation have also been reported in children and young adults.^24,25,26^ One study of 450 African American and Latino children and adolescents aged 8 to 18 years showed that exposure to traffic-related total NO_x_ and nonfreeway NO_x_ 1 year prior to the study visit was associated with higher HOMA-IR.^25^ Another study found that increased exposure to nitrogen dioxide 1 year prior to the study visit was associated with significantly higher insulin resistance in adolescents aged 15 years.^27^ However, some null associations have also been reported.^10,28^ For example, an earlier analysis of data from the CHS focusing on individuals aged 17 to 22 years did not find associations between prior 1-month and 1-year mean exposure to traffic-related total NO_x_ and insulin resistance markers.^10^ Overall, most existing studies examined short-term TRAP exposure (approximately 1 year) and have not consistently demonstrated clear links between TRAP and insulin resistance. Our study examined air pollution exposure over a longer duration (from pregnancy to early adolescence), providing evidence for a positive association between long-term childhood TRAP exposure and insulin resistance in later life.

Weight gain, a well-recognized risk factor for insulin resistance and T2D, has been found to be associated with both air pollution exposure^29^ and T2D risk. Previous studies found that the adverse effect of air pollution on glucose metabolism is more pronounced among obese children.^9,10^ However, it is important to note that overweight or obesity can manifest in early childhood, prior to onset of insulin resistance, which typically occurs after puberty.^30^ Adolescent BMI might be influenced by air pollution exposure early in life, before it contributes to the development of insulin resistance. To our knowledge, our study is among the first to disentangle the TRAP and insulin resistance link through a mediation angle. By using a fully lagged model, we found that nearly half of the total NO_x_–insulin resistance association can be explained by BMI growth trajectories.

Another notable contribution of our study was examining BMI growth trajectories for 10 years or longer, from early adolescence to young adulthood. Previous studies linked early childhood exposure to TRAP with BMI growth up to middle childhood.^31,32,33,34^ Earlier CHS studies found that traffic density and exposure to traffic-related NO_x_ in the 3 years prior to measurement were significantly associated with BMI growth from 5 to 11 years,^13^ and exposure to traffic-related NO_x_ from the prenatal period to the first year of life was associated with increased childhood BMI growth from 6 to 10 years.^14^ Another study of CHS participants reported that childhood exposure to traffic-related total NO_x_ and secondhand tobacco smoking had synergistic effects on accelerated BMI growth and attained BMI at 18 years of age.^12^ Overall, most previous studies reported a positive association between TRAP and BMI growth in childhood. Our study extended the examination of BMI trajectories through young adulthood and found that the association of childhood traffic-related NO_x_ exposure with BMI growth continues through young adulthood.

The mechanisms underlying these observations are not fully understood. Currently recognized mechanisms could involve systemic inflammation and disruption of metabolic pathways.^35^ The significant estimated mediation effect through BMI suggests that the inflammatory response triggered by air pollution could potentially disturb normal adipose tissue function, leading to abnormal lipid accumulation and weight gain.^36^ When adipose tissue reaches storage capacity, excess fat can be accumulated in the visceral organs such as the liver. Visceral fat may secrete inflammatory cytokines and adipokines, which can interfere with insulin signaling and contribute to insulin resistance.^8,37^

### Strengths and Limitations

Our study has considerable strengths. We conducted a thorough evaluation of childhood air pollution exposure from pregnancy to early adolescence. In our analytical framework, we ensured that the assessment of air pollution preceded the evaluation of BMI growth, and both occurred prior to the evaluation of adult insulin resistance. This allowed us to conduct a fully lagged life-course mediation model to accurately estimate the mediation role of BMI growth. Last, our study benefitted from long-term follow-up of CHS participants with repeated study measurements of BMI and evaluation of multiple measures of glucose dysregulation in young adulthood.

Despite these strengths, this study has some limitations. First, although the demographics of our sample were comparable with those of the larger cohort E, there might be unmeasured factors that could have influenced participants’ likelihood of participation. The generalization of our results should be limited to young populations living in urban areas with comparable demographic characteristics. Second, the relatively small sample size may limit our statistical power to conduct subgroup analysis (eg, by weight status). In one of our sensitivity analyses, the results found that the estimated mediating effect size of BMI among male participants was not statistically significant. While this observation may be statistically valid, we may lack statistical power to detect smaller differences. Future studies with larger sample sizes are encouraged to replicate our findings and further investigate associations within subgroups. Third, we were unable to fit a time-varying mediation model due to the unavailability of core variables between 15 and 24 years of age. Future research could explore susceptibility to air pollution throughout childhood. Last, the terms estimated *direct effects*, estimated *indirect effects*, estimated *mediation effects*, and estimated *total effects* used in this study refer to associations, rather than causal effects, and should be interpreted accordingly.^20^

## Conclusions

This cohort study found that nearly half of the total observed effect estimates of TRAP with insulin resistance were associated with accelerated BMI growth during the transition from early adolescence to young adulthood. This substantial percentage of observed estimated mediation effects emphasizes the essential need for managing weight during adolescence in reducing the risk of insulin resistance in young adulthood, particularly among children exposed to higher levels of traffic-related pollutants. Implementing preventive measures for weight control early in life may play a pivotal role in mitigating the impact of these environmental factors on insulin resistance later in life.

## Notes

Supplement 1. **eMethods.** California Line Source Dispersion Model (CALINE4) **eTable 1.** Estimated Direct, Indirect, and Total Effects of Childhood Exposure to Traffic-Related Air Pollution on Adult HOMA-IR, Stratified by Sex: Results From PROCESS Macro Model 6 **eTable 2.** Estimated Direct, Indirect, and Total Effects of Childhood Exposure to Traffic-Related Air Pollution on Adult HbA_1c_: Results From PROCESS Macro Model 6 (n = 276) **eTable 3.** Direct Paths Connecting Traffic-Related Exposures With HOMA-IR and HbA_1c_ **eFigure 1.** Flow Chart of Study Population **eFigure 2.** Measured BMI at 3 Time Points **eFigure 3.** Visual Representation of Relationships Between Study Variables (a Directed Acyclic Graph)
eReferences

Supplement 2.
Data Sharing Statement

## References

[1] Tahapary DL, Pratisthita LB, Fitri NA, . Challenges in the diagnosis of insulin resistance: focusing on the role of HOMA-IR and tryglyceride/glucose index. Diabetes Metab Syndr. 2022;16(8):102581. doi:10.1016/j.dsx.2022.102581 35939943

[2] Lascar N, Brown J, Pattison H, Barnett AH, Bailey CJ, Bellary S. Type 2 diabetes in adolescents and young adults. Lancet Diabetes Endocrinol. 2018;6(1):69-80. doi:10.1016/S2213-8587(17)30186-9 28847479

[3] Benson PE. CALINE 4—a dispersion model for predictiong air pollutant concentrations near roadways. November 1984. Accessed September 4, 2023. https://trid.trb.org/view/215944

[4] Rao X, Patel P, Puett R, Rajagopalan S. Air pollution as a risk factor for type 2 diabetes. Toxicol Sci. 2015;143(2):231-241. doi:10.1093/toxsci/kfu250 25628401 PMC4306726

[5] Thiering E, Cyrys J, Kratzsch J, . Long-term exposure to traffic-related air pollution and insulin resistance in children: results from the GINIplus and LISAplus birth cohorts. Diabetologia. 2013;56(8):1696-1704. doi:10.1007/s00125-013-2925-x 23666166 PMC3699704

[6] Parasin N, Amnuaylojaroen T, Saokaew S. Effect of air pollution on obesity in children: a systematic review and meta-analysis. Children (Basel). 2021;8(5):327. doi:10.3390/children8050327 33922616 PMC8146513

[7] Huang M, Chen J, Yang Y, Yuan H, Huang Z, Lu Y. Effects of ambient air pollution on blood pressure among children and adolescents: a systematic review and meta-analysis. J Am Heart Assoc. 2021;10(10):e017734. doi:10.1161/JAHA.120.017734 33942625 PMC8200690

[8] Hardy OT, Czech MP, Corvera S. What causes the insulin resistance underlying obesity? Curr Opin Endocrinol Diabetes Obes. 2012;19(2):81-87. doi:10.1097/MED.0b013e3283514e13 22327367 PMC4038351

[9] Qin J, Xia W, Liang G, . Association of fine particulate matter with glucose and lipid metabolism: a longitudinal study in young adults. *Occup Environ Med*. Published online February 26, 2021. doi:10.1136/oemed-2020-107039 33637624

[10] Kim JS, Chen Z, Alderete TL, . Associations of air pollution, obesity and cardiometabolic health in young adults: the Meta-AIR study. *Environ Int*. 2019;133(pt A):105180. doi:10.1016/j.envint.2019.105180 PMC6884139 31622905

[11] Gauderman WJ, Urman R, Avol E, . Association of improved air quality with lung development in children. N Engl J Med. 2015;372(10):905-913. doi:10.1056/NEJMoa1414123 25738666 PMC4430551

[12] McConnell R, Shen E, Gilliland FD, . A longitudinal cohort study of body mass index and childhood exposure to secondhand tobacco smoke and air pollution: the Southern California Children’s Health Study. Environ Health Perspect. 2015;123(4):360-366. doi:10.1289/ehp.1307031 25389275 PMC4384197

[13] Jerrett M, McConnell R, Wolch J, . Traffic-related air pollution and obesity formation in children: a longitudinal, multilevel analysis. Environ Health. 2014;13(1):49. doi:10.1186/1476-069X-13-49 24913018 PMC4106205

[14] Kim JS, Alderete TL, Chen Z, . Longitudinal associations of in utero and early life near-roadway air pollution with trajectories of childhood body mass index. Environ Health. 2018;17(1):64. doi:10.1186/s12940-018-0409-7 30213262 PMC6137930

[15] McConnell R, Berhane K, Yao L, . Traffic, susceptibility, and childhood asthma. Environ Health Perspect. 2006;114(5):766-772. doi:10.1289/ehp.8594 16675435 PMC1459934

[16] Farzan SF, Habre R, Danza P, . Childhood traffic-related air pollution and adverse changes in subclinical atherosclerosis measures from childhood to adulthood. Environ Health. 2021;20(1):44. doi:10.1186/s12940-021-00726-x 33853624 PMC8048028

[17] Guo F, Chen X, Howland S, . Perceived stress from childhood to adulthood and cardiometabolic end points in young adulthood: an 18-year prospective study. J Am Heart Assoc. 2024;13(3):e030741. doi:10.1161/JAHA.123.030741 38230530 PMC11056127

[18] Habre R, Girguis M, Urman R, . Contribution of tailpipe and non-tailpipe traffic sources to quasi-ultrafine, fine and coarse particulate matter in southern California. J Air Waste Manag Assoc. 2021;71(2):209-230. doi:10.1080/10962247.2020.1826366 32990509 PMC8112073

[19] Centers for Disease Control and Prevention. CDC extended BMI-for-age growth charts. December 21, 2022. Accessed July 18, 2023. https://www.cdc.gov/growthcharts/extended-bmi.htm

[20] Hayes AF. *Introduction to Mediation, Moderation, and Conditional Process Analysis, Second Edition: A Regression-Based Approach*. Guilford Publications; 2017.

[21] Hayes AF, Rockwood NJ. Regression-based statistical mediation and moderation analysis in clinical research: observations, recommendations, and implementation. Behav Res Ther. 2017;98:39-57. doi:10.1016/j.brat.2016.11.001 27865431

[22] Lurmann F, Avol E, Gilliland F. Emissions reduction policies and recent trends in Southern California’s ambient air quality. J Air Waste Manag Assoc. 2015;65(3):324-335. doi:10.1080/10962247.2014.991856 25947128 PMC5737709

[23] Andersen ZJ, Raaschou-Nielsen O, Ketzel M, . Diabetes incidence and long-term exposure to air pollution: a cohort study. Diabetes Care. 2012;35(1):92-98. doi:10.2337/dc11-1155 22074722 PMC3241311

[24] Dang J, Yang M, Zhang X, . Associations of exposure to air pollution with insulin resistance: a systematic review and meta-analysis. Int J Environ Res Public Health. 2018;15(11):2593. doi:10.3390/ijerph15112593 30463387 PMC6266153

[25] Toledo-Corral CM, Alderete TL, Habre R, . Effects of air pollution exposure on glucose metabolism in Los Angeles minority children. Pediatr Obes. 2018;13(1):54-62. doi:10.1111/ijpo.12188 27923100 PMC5722706

[26] Mann JK, Lutzker L, Holm SM, . Traffic-related air pollution is associated with glucose dysregulation, blood pressure, and oxidative stress in children. Environ Res. 2021;195:110870. doi:10.1016/j.envres.2021.110870 33587949 PMC8520413

[27] Thiering E, Markevych I, Brüske I, . Associations of residential long-term air pollution exposures and satellite-derived greenness with insulin resistance in German adolescents. Environ Health Perspect. 2016;124(8):1291-1298. doi:10.1289/ehp.1509967 26863688 PMC4977044

[28] Fleisch AF, Luttmann-Gibson H, Perng W, . Prenatal and early life exposure to traffic pollution and cardiometabolic health in childhood. Pediatr Obes. 2017;12(1):48-57. doi:10.1111/ijpo.12106 26843357 PMC4974151

[29] Huang C, Li C, Zhao F, Zhu J, Wang S, Sun G. The association between childhood exposure to ambient air pollution and obesity: a systematic review and meta-analysis. Int J Environ Res Public Health. 2022;19(8):4491. doi:10.3390/ijerph19084491 35457358 PMC9030539

[30] Jeffery AN, Metcalf BS, Hosking J, Streeter AJ, Voss LD, Wilkin TJ. Age before stage: insulin resistance rises before the onset of puberty: a 9-year longitudinal study (EarlyBird 26). Diabetes Care. 2012;35(3):536-541. doi:10.2337/dc11-1281 22279034 PMC3322712

[31] Jerrett M, McConnell R, Chang CCR, . Automobile traffic around the home and attained body mass index: a longitudinal cohort study of children aged 10-18 years. Prev Med. 2010;50(0 1)(suppl 1):S50-S58. doi:10.1016/j.ypmed.2009.09.026 19850068 PMC4334364

[32] Guo M, Xiao C, Yan H, . Association of air pollution exposure during gestational and the first year of life with physical growth in preschoolers. Int J Environ Health Res. 2023;33(4):337-347. doi:10.1080/09603123.2022.2029829 35098822

[33] Huang JV, Leung GM, Schooling CM. The association of air pollution with body mass index: evidence from Hong Kong’s “Children of 1997” birth cohort. Int J Obes (Lond). 2019;43(1):62-72. doi:10.1038/s41366-018-0070-9 29686381

[34] de Bont J, Díaz Y, de Castro M, . Ambient air pollution and the development of overweight and obesity in children: a large longitudinal study. Int J Obes (Lond). 2021;45(5):1124-1132. doi:10.1038/s41366-021-00783-9 33627774

[35] Rajagopalan S, Brook RD. Air pollution and type 2 diabetes: mechanistic insights. Diabetes. 2012;61(12):3037-3045. doi:10.2337/db12-0190 23172950 PMC3501850

[36] Ghigliotti G, Barisione C, Garibaldi S, . Adipose tissue immune response: novel triggers and consequences for chronic inflammatory conditions. Inflammation. 2014;37(4):1337-1353. doi:10.1007/s10753-014-9914-1 24823865 PMC4077305

[37] Schenk S, Saberi M, Olefsky JM. Insulin sensitivity: modulation by nutrients and inflammation. J Clin Invest. 2008;118(9):2992-3002. doi:10.1172/JCI34260 18769626 PMC2522344

