# Supplementary material for: Childhood Exposure to Air Pollution, Body Mass Index Trajectories, and Insulin Resistance Among Young Adults
Source: JAMA Netw Open. 2025 Apr 22;8(4):e256431. doi: 10.1001/jamanetworkopen.2025.6431 (PMC12015664; doi:10.1001/jamanetworkopen.2025.6431)
Supplement: Supplement 1. — eMethods. California Line Source Dispersion Model (CALINE4) eTable 1. Estimated Direct, Indirect, and Total Effects of Childhood Exposure to Traffic-Related Air Pollution on Adult HOMA-IR, Stratified by Sex: Results From PROCESS Macro Model 6 eTable 2. Estimated Direct, Indirect, and Total Effects of Childhood Exposure to Traffic-Related Air Pollution on Adult HbA1c: Results From PROCESS Macro Model 6 (n = 276) eTable 3. Direct Paths Connecting Traffic-Related Exposures With HOMA-IR and HbA1c eFigure 1. Flow Chart of Study Population eFigure 2. Measured BMI at 3 Time Points eFigure 3. Visual Representation of Relationships Between Study Variables (a Directed Acyclic Graph) eReferences [file jamanetwopen-e256431-s001.pdf]

Supplementary Online Content

Guo F, Chen X, Howland S, et al. Childhood exposure to air pollution, body mass index trajectories, and insulin resistance among young adults. *JAMA Netw Open.* 2025;8(4):e256431. doi:10.1001/jamanetworkopen.2025.6431

eMethods. California Line Source Dispersion Model (CALINE4)
eTable 1. Estimated Direct, Indirect, and Total Effects of Childhood Exposure to Traffic-Related Air Pollution on Adult HOMA-IR, Stratified by Sex: Results From PROCESS Macro Model 6
eTable 2. Estimated Direct, Indirect, and Total Effects of Childhood Exposure to Traffic-Related Air Pollution on Adult HbA_{1c}: Results From PROCESS Macro Model 6 (n = 276)
eTable 3. Direct Paths Connecting Traffic-Related Exposures With HOMA-IR and HbA_{1c}
eFigure 1. Flow Chart of Study Population
eFigure 2. Measured BMI at 3 Time Points
eFigure 3. Visual Representation of Relationships Between Study Variables (a Directed Acyclic Graph)
eReferences

This supplementary material has been provided by the authors to give readers additional information about their work.

© 2025 Guo F et al. *JAMA Network Open*.

eMethods. California Line Source Dispersion Model (CALINE4)

Supplemental Methods

California Line Source dispersion model (CALINE4)

We employed the California Line Source dispersion model (CALINE4) to estimate residential traffic-related NO_x concentrations from nearby on-road vehicles on freeways and highways (classified as Feature Class Codes (FCC)1 and FCC2 roads), non-freeways/highways (FCC3 and FCC4 roads) and their total.[1] The CALINE4 model is a reliable and effective tool for estimating vehicle emission concentrations at locations near roadways and freeways.[2,3] The model has been widely used in studies examining cardiometabolic health outcomes,[4–6] playing a key role in expanding our understanding of how traffic emissions affect health to the body of evidence regarding the health effects of traffic emissions.[7] CALINE4 provides high-resolution dispersion modeling, capturing fine-scale gradients in traffic-related air pollutants at specific receptor points, with variations over just a few meters. This model's outputs for tailpipe and non-tailpipe traffic emissions show a high level of correlation, indicating its effectiveness in capturing exposure to the entire mixture of near-roadway air pollutants. In a source apportionment analysis conducted on particular matter (PM) samples from eight CHS communities, including those in our study, CALINE4 freeway/highway NO_x consistently emerged as a strong predictor of both tailpipe and non-tailpipe contributions to PM across different size fractions such as $PM_{2.5–10}$ (coarse), $PM_{2.5}$ (fine), and $PM_{0.2}$ (quasi-ultrafine).[8]

© 2025 Guo F et al. *JAMA Network Open*.

eTable 1. Estimated Direct, Indirect, and Total Effects of Childhood Exposure to Traffic-Related Air Pollution on Adult HOMA-IR, Stratified by Sex: Results From PROCESS Macro Model 6

**Traffic related total NO_x**	**Female n=144**			**Male n=138**		
**Indirect effect**	**Beta**	**95% CI**[**]	**p value**	**Beta**	**95% CI**[**]	**p value**
$NO_x \rightarrow$ M1[††] $\rightarrow$ HOMA-IR	0.14	0.02, 0.31	--	0.04	-0.18, 0.29	--
$NO_x \rightarrow$ M2[‡‡] $\rightarrow$ HOMA-IR	0.09	-0.09, 0.36	--	-0.04	-0.31, 0.38	--
$NO_x \rightarrow$ M1 $\rightarrow$ M2 $\rightarrow$ HOMA-IR	0.04	-0.01, 0.15	--	0.01	-0.05, 0.10	--
**Total indirect effect**	0.28	0.25, 0.52	--	0.01	-0.33, 0.59	--
**Direct effect**						
$NO_x \rightarrow$ HOMA-IR	0.65	0.22, 0.08	0.003	-0.04	-0.40, 0.33	0.84
**Total effect**						
$NO_x \rightarrow$ HOMA-IR	0.93	0.48, 1.73	<0.001	-0.03	-0.53, 0.46	0.90

[**] Bootstrap confidence intervals were displayed for indirect effects.
[††] M1 represents the predicted individual BMI (intercept) at the age of 13 years.
[‡‡] M2 represents individual accelerated BMI growth (slope) from approximately 13 to 24 years old.

All analyses adjusted for adulthood age, race, ethnicity, participant smoking status, parents' highest degree, and parents' diabetes history.
Abbreviation: CI, confidence interval.

© 2025 Guo F et al. *JAMA Network Open*.

eTable 2. Estimated Direct, Indirect, and Total Effects of Childhood Exposure to Traffic-Related Air Pollution on Adult HbA_{1c}: Results From PROCESS Macro Model 6 (n = 276)

**Traffic related total NO_x**			
**Indirect effect**	**Beta**	**95% CI**[**]	**p value**
$NO_x \rightarrow$ M1[††] $\rightarrow$ HbA1c	0.018	-0.002, 0.05	--
$NO_x \rightarrow$ M2[‡‡] $\rightarrow$ HbA1c	0.002	-0.009, 0.02	--
$NO_x \rightarrow$ M1 $\rightarrow$ M2 $\rightarrow$ HbA1c	0.002	-0.001, 0.008	--
**Total indirect effect**	0.022	-0.0004, 0.06	--
**Direct effect**			
$NO_x \rightarrow$ HbA1c	0.062	0.01, 0.12	0.03
**Total effect**			
$NO_x \rightarrow$ HbA1c	0.084	0.03, 0.14	0.004
**Traffic density**			
**Indirect effect**	**Beta**	**95% CI**[**]	**p value**
Density $\rightarrow$ M1 $\rightarrow$ HbA1c	0.015	-0.0009, 0.05	--
Density $\rightarrow$ M2 $\rightarrow$ HbA1c	0.003	-0.006, 0.02	--
Density $\rightarrow$ M1 $\rightarrow$ M2 $\rightarrow$ HbA1c	0.002	-0.0008, 0.006	--
**Total indirect effect**	0.019	-0.0005, 0.06	--
**Direct effect**			
Traffic density $\rightarrow$ HbA1c	0.061	0.009, 0.114	0.02
**Total effect**			
Traffic density $\rightarrow$ HbA1c	0.080	0.03, 0.13	0.003

[**] Bootstrap confidence intervals were displayed for indirect effects.
[††] M1 represents the predicted individual BMI (intercept) at the age of 13 years.
[‡‡] M2 represents individual accelerated BMI growth (slope) from approximately 13 to 24 years old.
All analyses adjusted for adulthood age, sex, race, ethnicity, smoking status, parents' highest degree, and parents' diabetes history.
Abbreviation: CI, confidence interval.

© 2025 Guo F et al. *JAMA Network Open*.

eTable 3. Direct Paths Connecting Traffic-Related Exposures With HOMA-IR and HbA_{1c}

	HOMA-IR (n=282)			HbA1c (n=276)		
**Traffic related total NO_x**	**Beta**	**95% CI**	**p value**	**Beta**	**95% CI**	**p value**
NO_x → M1	0.71	0.29, 1.13	0.001	0.71	0.29, 1.14	0.001
NO_x → M2	0.01	-0.04, 0.07	0.617	0.01	-0.04, 0.07	0.64
M1 → M2	0.02	0.01, 0.04	0.002	0.03	0.01, 0.04	0.001
M1 → outcome	0.21	0.14, 0.29	<0.001	0.03	0.01, 0.04	0.002
M2 → outcome	2.61	2.01, 3.21	<0.001	0.11	-0.01, 0.24	0.07
**Traffic density**	**Beta**	**95% CI**	**p value**	**Beta**	**95% CI**	**p value**
Density → M1	0.56	0.16, 0.96	0.006	0.58	0.18, 0.98	0.005
Density → M2	0.03	-0.03, 0.08	0.318	0.02	-0.03, 0.07	0.39
M1 → M2	0.02	0.009, 0.04	0.002	0.03	0.01, 0.04	0.001
M1→ outcome	0.22	0.14, 0.30	<0.001	0.03	0.01, 0.04	0.002
M2→ outcome	2.60	1.99, 3.20	<0.001	0.11	-0.01, 0.24	0.08

M1 represents the predicted individual BMI (intercept) at the age of 13 years
M2 represents individual accelerated BMI growth (slope) from approximately 13 to 24 years old
All analyses adjusted for adulthood age, sex, race, ethnicity, participant smoking status, parents' highest degree, and parents' diabetes history.

© 2025 Guo F et al. *JAMA Network Open*.

eFigure 1. Flow Chart of Study Population

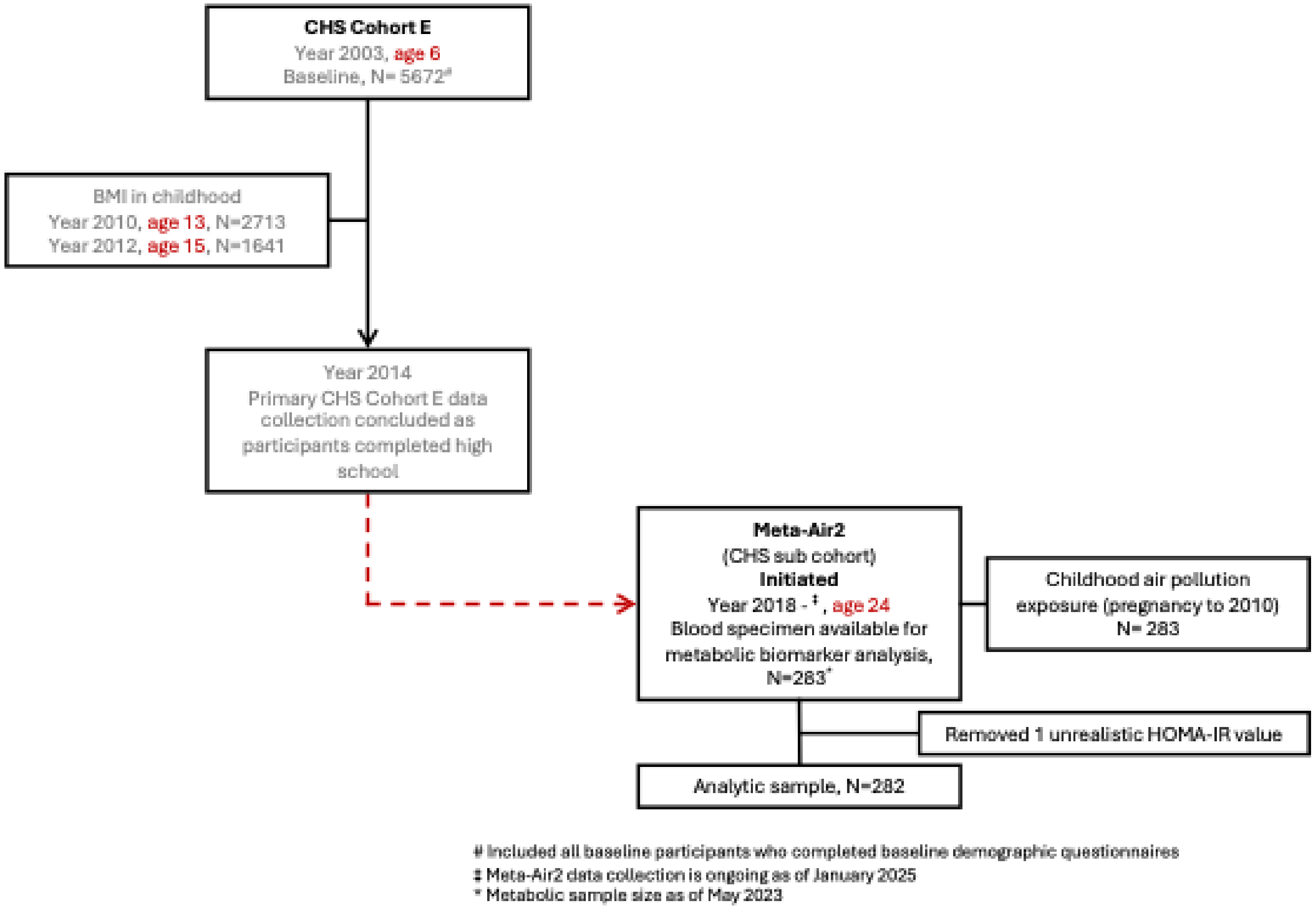

© 2025 Guo F et al. *JAMA Network Open*.

eFigure 2. Measured BMI at 3 Time Points

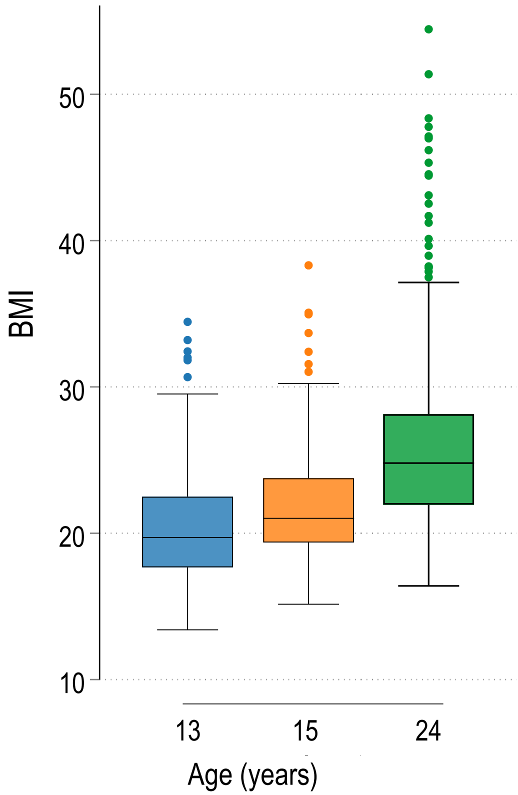

Age (years)	N	Measured BMI
13.4 ± 0.6	250	20.5 ± 3.8
15.1 ± 0.6	211	21.9 ± 3.8
24.0 ± 1.7	282	26.2 ± 6.5

© 2025 Guo F et al. *JAMA Network Open*.

eFigure 3. Visual Representation of Relationships Between Study Variables (a Directed Acyclic Graph)

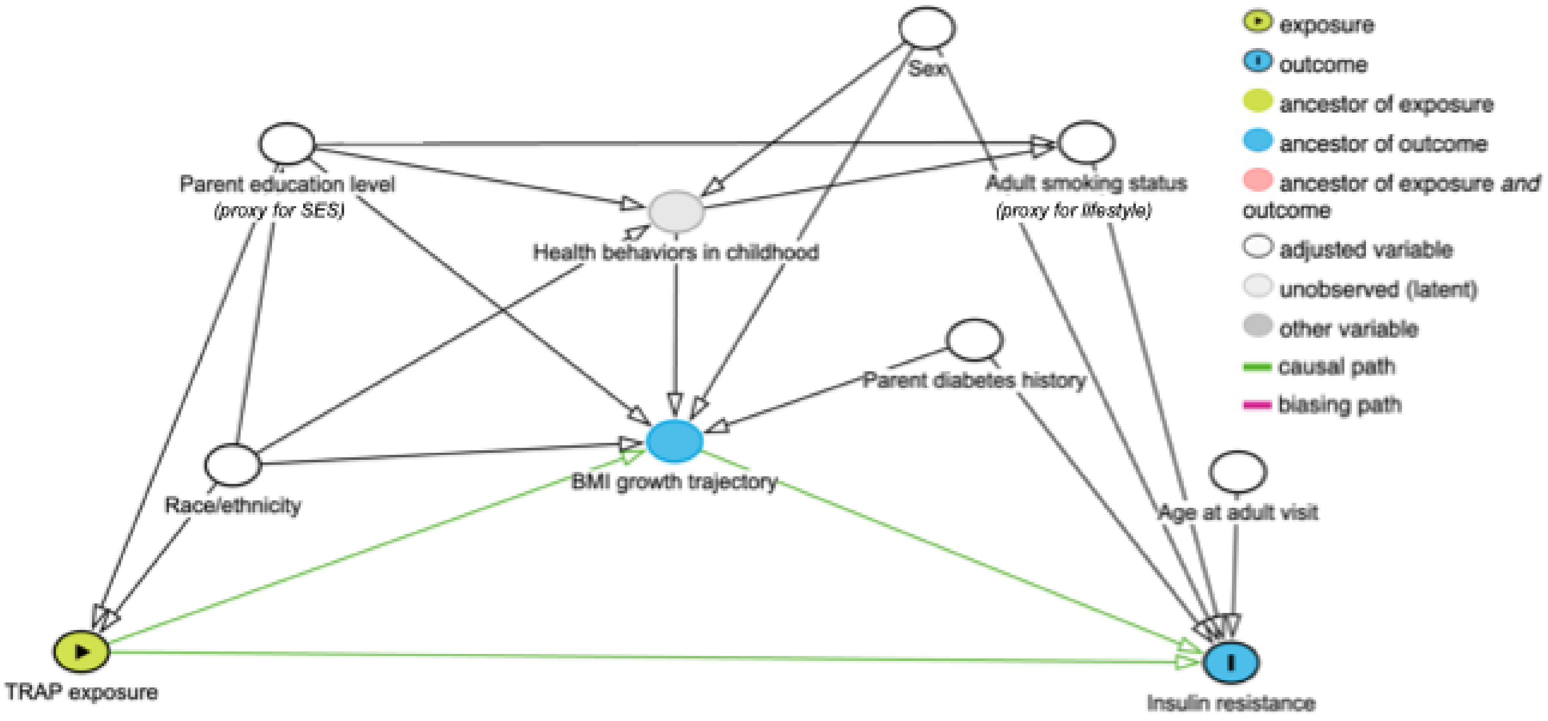

© 2025 Guo F et al. *JAMA Network Open*.

eReferences

1. Benson PE. CALINE 4-A DISPERSION MODEL FOR PREDICTIONG AIR POLLUTANT CONCENTRATIONS NEAR ROADWAYS. Published online November 1984. Accessed September 4, 2023. https://trid.trb.org/view/215944
2. Franklin M, Vora H, Avol E, et al. Predictors of intra-community variation in air quality. *J Expo Sci Environ Epidemiol*. 2012;22(2):135-147. doi:10.1038/jes.2011.45
3. Fruin SA, Hudda N, Sioutas C, Delfino RJ. Predictive Model for Vehicle Air Exchange Rates Based on a Large, Representative Sample. *Environ Sci Technol*. 2011;45(8):3569-3575. doi:10.1021/es103897u
4. Kim JS, Chen Z, Alderete TL, et al. Associations of air pollution, obesity and cardiometabolic health in young adults: The Meta-AIR study. *Environ Int*. 2019;133(Pt A):105180. doi:10.1016/j.envint.2019.105180
5. Jerrett M, McConnell R, Wolch J, et al. Traffic-related air pollution and obesity formation in children: a longitudinal, multilevel analysis. *Environmental Health*. 2014;13(1):49. doi:10.1186/1476-069X-13-49
6. Farzan SF, Habre R, Danza P, et al. Childhood traffic-related air pollution and adverse changes in subclinical atherosclerosis measures from childhood to adulthood. *Environ Health*. 2021;20(1):44. doi:10.1186/s12940-021-00726-x
7. Institute HE. State of Global Air 2019: Air pollution a significant risk factor worldwide. Health Effects Institute. June 7, 2019. Accessed October 6, 2023. https://www.healtheffects.org/announcements/state-global-air-2019-air-pollution-significant-risk-factor-worldwide
8. Habre R, Girguis M, Urman R, et al. Contribution of tailpipe and non-tailpipe traffic sources to quasi-ultrafine, fine and coarse particulate matter in southern California. *J Air Waste Manag Assoc*. 2021;71(2):209-230. doi:10.1080/10962247.2020.1826366

© 2025 Guo F et al. *JAMA Network Open*.
