# Supplementary material for: Childhood Exposure to Air Pollution, Body Mass Index Trajectories, and Insulin Resistance Among Young Adults
Source: JAMA Netw Open. 2025 Apr 22;8(4):e256431. doi: 10.1001/jamanetworkopen.2025.6431 (PMC12015664; doi:10.1001/jamanetworkopen.2025.6431)
Supplement: Supplement 2. — Data Sharing Statement [file jamanetwopen-e256431-s002.pdf]

Data Sharing Statement

Guo. Childhood Exposure to Air Pollution, Body Mass Index Trajectories, and Insulin Resistance Among Young Adults. *JAMA Netw Open*. Published April 22, 2025. doi:10.1001/jamanetworkopen.2025.6431

Data
Data available: Yes
Data types: Data dictionary
How to access data: Investigators who require access to data will be required to submit a research protocol, which will be reviewed by a committee of CHS investigators and the USC IRB. This Committee will judge the scientific validity of the request, with the sole aim of guarding against inappropriate use. If the study is deemed scientifically valid, the Committee will provide the requested data pending assurances that the confidentiality of our study subjects will not be violated. Any data provided will be stripped of specific subject identifiers, including name, address, geographic coordinates, work locations, dates, and any other data that could be used to identify a specific participant or his/her residence. Recipients must agree to security policies, including password-protected access and encrypted storage that will minimize the risk of unauthorized distribution. All data sharing proposals will be reviewed and approved by the USC IRB.
When available: With publication

Supporting Documents
Document types: None

Additional Information
Who can access the data: Investigators who require access to data will be required to submit a research protocol, which will be reviewed by a committee of CHS investigators and the USC IRB.
Types of analyses: If the study is deemed scientifically valid, the Committee will provide the requested data pending assurances that the confidentiality of our study subjects will not be violated.
Mechanisms of data availability: This Committee will judge the scientific validity of the request, with the sole aim of guarding against inappropriate use. If the study is deemed scientifically valid, the Committee will provide the requested data pending assurances that the confidentiality of our study subjects will not be violated. Any data provided will be stripped of specific subject identifiers, including name, address, geographic coordinates, work locations, dates, and any other data that could be used to identify a specific participant or his/her residence. Recipients must agree to security policies, including password-protected access and encrypted storage that will minimize the risk of unauthorized distribution. All data sharing proposals will be reviewed and approved by the USC IRB.
